# Association of vitamin D pathway genes polymorphisms with pulmonary tuberculosis susceptibility in a Chinese population

**DOI:** 10.1186/s12263-021-00687-3

**Published:** 2021-04-21

**Authors:** Tian-Ping Zhang, Shuang-Shuang Chen, Gen-You Zhang, Si-Jiu Shi, Li Wei, Hong-Miao Li

**Affiliations:** 1 The First Affiliated Hospital of USTC, Division of Life Sciences and Medicine, University of Science and Technology of China, 17 Lujiang Road, Hefei, Anhui 230001 People’s Republic of China; 2 Anhui Chest Hospital (Anhui Provincial TB Institute), Clinical College of Chest, Anhui Medical University, 397 Jixi Road, Hefei, Anhui 230022 People’s Republic of China; 3 Pharmacoepidemiology and Medication Safety Research Cluster, UCL School of Pharmacy, London, UK

**Keywords:** Pulmonary tuberculosis, Infectious disease, Mycobacterium tuberculosis, Vitamin D pathway, Single nucleotide polymorphisms

## Abstract

**Objective:**

This study aimed to evaluate the association of single nucleotide polymorphisms (SNPs) of vitamin D metabolic pathway genes with susceptibility to pulmonary tuberculosis (PTB).

**Methods:**

Nine hundred seventy-nine patients (490 PTB cases and 489 healthy controls) were included in this study. Seventeen SNPs of vitamin D metabolic pathway genes, including *CYP24A1*, *CYP27A1*, *CYP27B1*, *CYP2R1*, *GC*, and *DHCR7*, were genotyped with improved multiple ligase detection reaction (iMLDR).

**Results:**

The *GC* rs3733359 GA, rs16847024 CT genotypes were significantly associated with the reduced risk of PTB, and the rs3733359 A, rs16847024 T alleles were also associated with the decreased PTB susceptibility. The GT genotype of *GC* rs4588 variant was significantly higher in patients with PTB when compared to controls. Moreover, the increased risk of rs3733359 and rs16847024 variants, and a decreased risk of rs4588, were found under the dominant mode among the PTB patients. However, there was no significant relationship of *CYP24A1*, *CYP27A1*, *CYP27B1*, *CYP2R1*, and *DHCR7* polymorphisms with the risk of PTB. In *CYP27A1*, the rs17470271 T and rs933994 T alleles were significantly associated with leukopenia, drug resistance in the PTB patients, respectively. In *GC* gene, the rs7041 and rs3733359 variants were found to be associated with pulmonary infection, fever in the PTB patients, respectively. The increased frequency of rs16847024 TT genotype was found in the PTB patients with fever and drug-induced liver damage. *DHCR7* rs12785878 TT genotype, and T allele frequencies were both significantly associated with pulmonary infection in the PTB patients. The haplotype analysis showed that *CYP24A1* TACT, *CYP2R1* GGCT, GGAT, *GC* AATG haplotypes were related to PTB susceptibility.

**Conclusion:**

Our study suggested that *GC* SNPs were associated with the genetic background of PTB. *CYP27A1*, *GC*, and *DHCR7* genetic variations might contribute to several clinical phenotypes of PTB in Chinese.

**Supplementary Information:**

The online version contains supplementary material available at 10.1186/s12263-021-00687-3.

## Introduction

Pulmonary tuberculosis (PTB), which is caused by the human pathogen *Mycobacterium tuberculosis* (MTB), continues to be the leading cause of morbidity and mortality worldwide [1]. The latest World Health Organization (WHO) figures showed that there were about 10.0 million new TB patients globally in 2018. Of them, 0.87 million cases were from China [2]. Currently, PTB poses a major public health challenge and is a significant economic burden in Asian region; hence, it is important to identify factors that increase disease susceptibility in order to provide evidence for effective control strategies. Cell-mediated immunity is essential for inhibiting MTB infection, regulating the first defense against MTB, and has an important role in the development of PTB [3]. In addition, previous studies had shown that only 10% of these people infected with MTB finally developed active PTB, and the identical twins were twice as likely to progress to this disease as fraternal twins [4, 5]. These facts supported the viewpoint that susceptibility to PTB upon MTB infection was affected by the host genetic and environmental factors. Variation of many genes had been identified to be associated with genetic susceptibility to PTB in previous studies [6–8].

The existing evidence showed that the vitamin D metabolic pathway might be implicated to the pathogenesis of PTB. Vitamin D deficiency was much more common in patients with PTB, and the serum vitamin D expression was negatively related to disease severity [9, 10]. Another study found that there was ethnic difference in the 25-hydroxyvitamin D (25(OH)D) level among the new TB cases, and further studies confirmed that vitamin D deficiency resulted in reduced immunity against bacteria, and increased the risk of TB [11–13]. 1-25-dihydroxyvitamin D3 (1,25(OH)2D3), known as the active form of vitamin D, is a key hormone that regulated the activity of different defense and immune cells, including epithelial, lymphocytes, macrophages, and monocytes cells [14]. 1,25(OH)2D3 has also been reported to increase innate immunity through enhancing the antimicrobial peptides expression, and promoting the autophagy of infected cells, thereby restraining the MTB growth in macrophages cells [15]. Immune modulation by vitamin D included enhancing innate immune response, attenuating and stimulating Th1 and Th2 cell proliferation, respectively, and hypovitaminosis D was associated with autoimmune diseases, Alzheimer’s disease, etc. [16, 17]. Hence, the influence of the individual variations in vitamin D metabolism on the immune responses, patients should not be neglected in patients with PTB.

Many genes associated with vitamin D metabolic pathway were involved in the host susceptibility to persistent TB infection in which vitamin D receptor (VDR) had been well studied [18–20]. Vitamin D exerted its biological functions through binding with VDR; hence, VDR variant might contribute to the TB development by resulting in diminished function of vitamin D. The study by Hu et al. demonstrated a significant association between *VDR* rs11574143, rs11168287, rs11574079 polymorphisms and PTB susceptibility in the Chinese population [21]. Besides VDR, other genes involved in vitamin D pathway, including *CYP24A1*, *CYP27A1*, *CYP27B1*, *CYP2R1*, *GC*, *DHCR7*, might also be associated with TB susceptibility [22]. It is very meaningful to study the influence of genetic variation of multiple genes in the context of vitamin D metabolic pathway, rather than a single gene, in the development of PTB. However, there were no studies to analyze the relationship of the collective vitamin D metabolic pathway genes and PTB susceptibility in a Chinese population. Thus, this study was designed to evaluate the associations of single nucleotide polymorphisms (SNPs) of vitamin D pathway genes (*CYP24A1*, *CYP27A1*, *CYP27B1*, *CYP2R1*, *GC*, *DHCR7*) with PTB susceptibility in a Chinese population.

## Materials and methods

### Study participants

In this case-control study, we recruited 500 patients with PTB from the Department of Tuberculosis at Anhui Chest Hospital during the period of April to October 2019. The PTB patients were diagnosed by a specialist according to the following criteria: suspicious clinical symptoms, chest radiography, sputum and/or bronchoalveolar lavage fluid MTB culture, microscopy for acid fast bacilli (AFB), and effect of anti-TB treatment. We excluded PTB patients with HIV positive, hepatitis, malignant tumor, and immune-compromised conditions. We enrolled a random sample of 500 unrelated healthy individuals without a history of TB, malignant tumor, and HIV, from health examine center in the same area to serve as controls. All controls needed to be asymptomatic with negative sputum smear and culture, and normal chest radiograph.

Our study was approved by the Ethics Committee of Anhui Chest Hospital (K2020-005). We collected peripheral blood samples and other information from the study participants after obtaining an informed consent from each participant. The information included age, gender, and clinical data of PTB, such as fever, drug resistance, drug-induced liver damage (DILI), pulmonary infection, leukopenia, and sputum smear*.*

### DNA extraction

Approximately 5 mL peripheral blood samples were drawn by tubes containing ethylenediaminetetraacetic acid with median cubital vein, and stored at −20 °C for DNA extraction. Then, we extracted genomic DNA from the peripheral blood leukocytes according to the standard procedures of the Flexi Gene-DNA Kit (Qiagen, Valencia, CA).

### SNP selection and genotyping

Six genes of the vitamin D metabolic pathway, including *CYP24A1*, *CYP27A1*, *CYP27B1*, *CYP2R1*, *GC*, *DHCR7*, were included in this study for analyses. We systematically searched the existing literature on the association of the polymorphisms in vitamin D metabolic pathway genes with human diseases, and looked for the SNPs associated with human disease. Then, we obtained the genotype data on these six genes of Han Chinese people in Beijing with the Ensembl Genome Browser 85 and CHBS_1000g, and used the pairwise option of the HaploView 4.0 software (Cambridge, MA, USA) for selecting the tag SNPs of each gene. Finally, we used SNP function prediction to assess the potential function of the relevant SNPs. In conclusion, we selected four SNPs (rs2248359, rs2296241, rs927650, rs6013897) of *CYP24A1*, two SNPs (rs17470271, rs933994) of *CYP27A1*, one SNPs (rs4646536) of *CYP27B1*, four SNPs (rs12794714, rs10741657, rs7935792, rs1562902) of *CYP2R1*, four SNPs (rs7041, rs3733359, rs16847024, rs4588) of *GC*, two SNPs (rs12785878, rs3829251) of *DHCR7*, respectively. All SNPs satisfied the following two conditions: minor allele frequency (MAF) ≥ 0.05 in CHB, *r*^*2*^ threshold > 0.8.

This study adopted improved multiple ligase detection reaction (iMLDR) genotyping assay for genotyping with the technical support of the Center for Genetic and Genomic Analysis, Genesky Biotechnologies (Inc., Shanghai). Only the individual with 100% genotyping success rate for all SNPs was included in the final analysis.

### Statistical analysis

We used the Epi Data 3.1 software to enter the data, and performed the statistical analysis by SPSS 23.0 (SPSS Inc., IL, USA). Hardy-Weinberg equilibrium test of each SNP frequency among normal controls was evaluated by Chi-square. The genotype, allele frequencies differences of all SNPs between the PTB patients and normal controls were assessed by Chi-square test, and odds ratios (OR), 95% confidence intervals (CI) using the logistic regression models. We also investigated the associations between SNPs and the risk for PTB in two genetic models (dominant, recessive model), and used the SHEsis software to conduct the haplotype analysis [23]. All of the *P* values presented were two-sided, and *P* < 0.05 was considered as the threshold of statistical significance. The Bonferroni correction was used for multiple testing in SNP analysis.

## Results

Our study finally included 490 PTB patients and 489 normal controls. The PTB group included 172 females and 318 males, with a mean age of 44.92±17.77, while 265 females and 224 males were enrolled in controls with a mean age of 43.43±12.95. The results showed that all the SNPs were conformed to Hardy Weinberg equilibrium in controls. The proportion of several common clinical features in patients with PTB were sputum smear-positive (26.73%), pulmonary infection (21.63%), fever (17.75%), drug resistance (15.31%), DILI (14.29%), and leukopenia (6.73%), respectively.

### Association of vitamin D pathway genes SNPs with PTB

The results of allele and genotype frequencies of all the SNPs in *CYP24A1*, *CYP27A1*, *CYP27B1*, *CYP2R1*, *GC*, and *DHCR7* genes are summarized in Table 1. We noted that the frequencies of *GC* rs3733359 GA genotype, rs16847024 CT genotype, rs3733359 A allele, and rs16847024 T allele were significantly decreased in PTB patients when compared with the normal controls (GA versus GG: *P* = 0.006; CT versus CC: *P* = 0.003, A versus G: *P* = 0.029; T versus C: *P* = 0.020, respectively). However, the GT genotype frequency of *GC* rs4588 variant in PTB patients was significantly higher than that in controls (GT versus GG: *P* = 0.006). Moreover, the increased risk of rs3733359, rs16847024 variants, and a decreased risk of rs4588, were found under the dominant mode (GG versus AA+GA: *P* = 0.006; CC versus TT+CT: *P* = 0.006; GG versus TT+GT: *P* = 0.019, respectively). *GC* rs7041 variant was not associated with PTB susceptibility.

**Table 1:** Genotypes and alleles frequencies of vitamin D pathway genes in PTB patients and normal controls

SNP	Analyze model		PTB (*N* = 490) *n* (%)	Control (*N* = 489) *n* (%)	*P* value ^*^	*OR* (95% CI)
*CYP24A1*						
rs2248359	Genotypes	TT	73 (14.90)	66 (13.50)	0.660	0.941 (0.717, 1.235)
		CT	235 (47.96)	232 (47.44)	0.454	0.862 (0.583, 1.272)
		CC	182 (37.14)	191 (39.06)	Reference	
	Alleles	T	381 (38.88)	364 (37.22)	0.450	0.932 (0.777, 1.119)
		C	599 (61.12)	614 (62.78)	Reference	
	Dominant model	CC	182 (37.14)	191 (39.06)	0.537	1.031 (0.935, 1.138)
		CT+TT	308 (62.86)	298 (60.94)	Reference	
	Recessive model	TT	73 (14.90)	66 (13.50)	0.530	0.984 (0.935, 1.035)
		CT+CC	417 (85.10)	423 (86.50)	Reference	
rs2296241	Genotypes	AA	81 (16.53)	83 (16.97)	0.972	1.007 (0.693, 1.462)
		GA	242 (49.39)	236 (48.26)	0.763	0.958 (0.725, 1.266)
		GG	167 (34.08)	170 (34.77)	Reference	
	Alleles	A	404 (41.22)	402 (41.10)	0.957	1.003 (0.902, 1.115)
		G	576 (58.78)	576 (58.90)	Reference	
	Dominant model	GG	167 (34.08)	170 (34.77)	0.822	0.980 (0.825, 1.165)
		AA+GA	323 (65.92)	319 (65.23)	Reference	
	Recessive model	AA	81 (16.53)	83 (16.97)	0.853	1.005 (0.950, 1.063)
		GG+GA	409 (83.47)	406 (83.03)	Reference	
rs927650	Genotypes	TT	37 (7.55)	32 (6.54)	0.704	0.950 (0.729, 1.238)
		TC	186 (37.96)	182 (37.22)	0.495	0.840 (0.508, 1.388)
		CC	267 (54.59)	275 (56.24)	Reference	
	Alleles	T	260 (26.53)	246 (25.15)	0.486	0.982 (0.932, 1.034)
		C	720 (73.47)	732 (74.85)	Reference	
	Dominant model	CC	267 (54.48)	275 (56.24)	0.582	1.040 (0.904, 1.196)
		TT+TC	223 (45.51)	214 (43.76)	Reference	
	Recessive model	TT	37 (7.55)	32 (6.54)	0.538	0.989 (0.956, 1.024)
		CC+TC	453 (92.45)	457 (93.46)	Reference	
rs6013897	Genotypes	AA	8 (1.63)	15 (3.07)	1.150	1.897 (0.794, 4.532)
		TA	135 (27.55)	131 (26.79)	0.898	0.982 (0.740, 1.303)
		TT	347 (70.82)	343 (70.14)	Reference	
	Alleles	A	151 (15.41)	161 (16.46)	0.524	1.013 (0.974, 1.052)
		T	829 (84.59)	817 (83.54)	Reference	
	Dominant model	TT	347 (70.82)	343 (70.14)	0.817	0.977 (0.805, 1.186)
		AA+TA	143 (29.18)	146 (29.86)	Reference	
	Recessive model	AA	8 (1.63)	15 (3.07)	0.138	1.015 (0.995, 1.035)
		TT+TA	482 (98.37)	474 (96.93)	Reference	
*CYP27A1*						
rs17470271	Genotypes	TT	2 (0.41)	6 (1.23)	0.407	0.861 (0.605, 1.225)
		AT	78 (15.92)	68 (13.91)	0.185	2.964 (0.595, 14.770)
		AA	410 (83.67)	415 (84.87)	Reference	
	Alleles	T	82 (8.37)	80 (8.18)	0.880	0.998 (0.972, 1.025)
		A	898 (91.63)	898 (91.82)	Reference	
	Dominant model	AA	410 (83.67)	415 (84.87)	0.608	1.079 (0.807, 1.442)
		TT+AT	80 (16.33)	74 (15.13)	Reference	
	Recessive model	TT	2 (0.41)	6 (1.23)	0.155	0.333 (0.067, 1.640)
		AA+AT	488 (99.59)	483 (98.77)	Reference	
rs933994	Genotypes	TT	17 (3.47)	10 (2.05)	0.159	0.565 (0.255, 1.251)
		CT	132 (26.94)	124 (25.36)	0.482	0.902 (0.677, 1.202)
		CC	341 (69.59)	355 (72.60)	Reference	
	Alleles	T	166 (16.94)	144 (14.72)	0.179	0.974 (0.937, 1.012)
		C	814 (83.06)	834 (85.28)	Reference	
	Dominant model	CC	341 (69.59)	355 (72.60)	0.300	1.110 (0.911, 1.351)
		TT+CT	149 (30.41)	134 (27.40)	Reference	
	Recessive model	TT	17 (3.47)	10 (2.05)	0.174	0.985 (0.965, 1.006)
		CC+CT	473 (96.53)	479 (97.95)	Reference	
rs4646536	Genotypes	AA	65 (13.27)	67 (13.70)	0.851	0.963 (0.651, 1.425)
		AG	226 (46.12)	209 (42.74)	0.288	0.864 (0.660, 1.131)
		GG	199 (40.61)	213 (43.56)	Reference	
	Alleles	A	356 (36.33)	343 (35.07)	0.562	0.981 (0.918, 1.048)
		G	624 (63.67)	635 (64.93)	Reference	
	Dominant model	GG	199 (40.61)	213 (43.56)	0.351	1.052 (0.946, 1.171)
		AA+AG	291 (59.39)	276 (56.44)	Reference	
	Recessive model	AA	65 (13.27)	67 (13.70)	0.842	0.968 (0.705, 1.330)
		GG+AG	425 (86.73)	422 (86.30)	Reference	
*CYP2R1*						
rs12794714	Genotypes	AA	67 (13.67)	55 (11.25)	0.222	0.775 (0.514, 1.167)
		GA	239 (48.78)	239 (48.88)	0.673	0.944 (0.721, 1.236)
		GG	184 (37.55)	195 (39.88)	Reference	
	Alleles	A	373 (38.06)	349 (35.69)	0.276	0.963 (0.900, 1.031)
		G	607 (61.94)	629 (64.31)	Reference	
	Dominant model	GG	184 (37.55)	195 (39.88)	0.455	1.039 (0.940, 1.147)
		AA+GA	306 (62.45)	294 (60.12)	Reference	
	Recessive model	AA	67 (13.67)	55 (11.25)	0.251	0.973 (0.928, 1.020)
		GG+GA	423 (86.33)	434 (88.75)	Reference	
rs10741657	Genotypes	AA	72 (14.69)	68 (13.91)	0.856	1.036 (0.704, 1.526)
		AG	215 (43.88)	236 (48.26)	0.180	1.204 (0.918, 1.581)
		GG	203 (41.43)	185 (37.83)	Reference	
	Alleles	A	359 (36.63)	372 (38.04)	0.521	1.023 (0.955, 1.095)
		G	621 (63.37)	606 (61.96)	Reference	
	Dominant model	GG	203 (41.43)	185 (37.83)	0.250	0.942 (0.851, 1.043)
		AA+AG	287 (58.57)	304 (62.17)	Reference	
	Recessive model	AA	72 (14.69)	68 (13.91)	0.725	1.057 (0.777, 1.436)
		GG+AG	418 (85.31)	421 (86.09)	Reference	
rs7935792	Genotypes	CC	10 (2.04)	6 (1.23)	0.286	0.574 (0.206, 1.594)
		AC	111 (22.65)	97 (19.84)	0.252	0.835 (0.614, 1.136)
		AA	369 (75.31)	386 (78.94)	Reference	
	Alleles	C	131 (13.67)	109 (11.15)	0.134	0.975 (0.943, 1.008)
		A	849 (86.63)	869 (88.85)	Reference	
	Dominant model	AA	369 (75.31)	386 (78.94)	0.176	1.172 (0.931, 1.477)
		CC+AC	121 (24.69)	103 (21.06)	Reference	
	Recessive model	CC	10 (2.04)	6 (1.23)	0.315	0.992 (0.976, 1.008)
		AA+AC	480 (97.96)	483 (98.77)	Reference	
rs1562902	Genotypes	CC	84 (17.14)	86 (17.59)	0.799	1.049 (0.724, 1.521)
		CT	242 (49.39)	243 (49.69)	0.841	1.029 (0.777, 1.364)
		TT	164 (33.47)	160 (32.72)	Reference	
	Alleles	C	410 (41.84)	415 (42.43)	0.789	1.010 (0.937, 1.090)
		T	570 (58.16)	563 (57.57)	Reference	
	Dominant model	TT	164 (33.47)	160 (32.72)	0.803	0.989 (0.905, 1.080)
		CC+CT	326 (66.53)	329 (67.28)	Reference	
	Recessive model	CC	84 (17.14)	86 (17.59)	0.854	1.005 (0.949, 1.065)
		TT+CT	406 (82.86)	403 (82.41)	Reference	
*GC*						
rs7041	Genotypes	CC	23 (4.69)	31 (6.34)	0.203	1.443 (0.821, 2.537)
		AC	178 (36.33)	188 (38.45)	0.362	1.131 (0.868, 1.472)
		AA	289 (58.98)	270 (55.22)	Reference	
	Alleles	C	224 (22.86)	250 (25.56)	0.162	1.036 (0.986, 1.090)
		A	756 (77.14)	728 (74.44)	Reference	
	Dominant model	AA	289 (58.98)	270 (55.22)	0.234	0.916 (0.792, 1.059)
		CC+AC	201 (41.02)	219 (44.78)	Reference	
	Recessive model	CC	23 (4.69)	31 (6.34)	0.259	1.018 (0.987, 1.049)
		AA+AC	467 (95.31)	458 (93.66)	Reference	
rs3733359	Genotypes	AA	50 (10.20)	53 (10.84)	0.238	1.296 (0.843, 1.994)
		GA	204 (41.63)	243 (49.69)	0.006	1.457 (1.116, 1.901)
		GG	236 (48.16)	193 (39.47)	Reference	
	Alleles	A	304 (31.02)	349 (35.69)	0.029	1.073 (1.007, 1.142)
		G	676 (68.98)	629 (64.31)	Reference	
	Dominant model	GG	236 (48.16)	193 (39.47)	0.006	0.856 (0.766, 0.957)
		AA+GA	254 (51.84)	296 (60.53)	Reference	
	Recessive model	AA	50 (10.20)	53 (10.84)	0.746	1.007 (0.985, 1.051)
		GG+GA	440 (89.80)	436 (89.16)	Reference	
rs16847024	Genotypes	TT	13 (2.65)	11 (2.25)	0.889	0.943 (0.417, 2.134)
		CT	99 (20.20)	139 (28.43)	0.003	1.566 (1.164, 2.106)
		CC	378 (77.14)	339 (69.32)	Reference	
	Alleles	T	125 (12.76)	161 (16.46)	0.020	0.775 (0.624, 0.962)
		C	855 (87.24)	817 (83.54)	Reference	
	Dominant model	CC	378 (77.14)	339 (69.32)	0.006	1.113 (1.031, 1.201)
		TT+CT	112 (22.86)	150 (30.68)	Reference	
	Recessive model	TT	13 (2.65)	11 (2.25)	0.583	0.996 (0.976, 1.016)
		CC+CT	477 (97.35)	478 (97.75)	Reference	
rs4588	Genotypes	TT	43 (8.78)	53 (10.84)	0.873	1.037 (0.664, 1.620)
		GT	256 (52.24)	209 (42.74)	0.006	0.687 (0.527, 0.896)
		GG	191 (38.98)	227 (46.42)	Reference	
	Alleles	T	342 (34.90)	315 (32.21)	0.208	1.083 (0.956, 1.228)
		G	638 (65.10)	663 (67.79)	Reference	
	Dominant model	GG	191 (38.98)	227 (46.42)	0.019	0.840 (0.726, 0.972)
		TT+GT	299 (61.02)	262 (53.58)	Reference	
	Recessive model	TT	143 (29.18)	53 (10.84)	0.278	1.023 (0.982, 1.066)
		GG+GT	447 (91.22)	436 (89.16)	Reference	
*DHCR7*						
rs12785878	Genotypes	TT	108 (22.04)	102 (20.86)	0.814	1.044 (0.730, 1.493)
		GT	235 (47.96)	254 (51.94)	0.236	1.195 (0.890, 1.603)
		GG	147 (30.00)	133 (27.20)	Reference	
	Alleles	T	451 (46.02)	458 (46.83)	0.719	1.015 (0.935, 1.103)
		G	529 (53.98)	520 (53.17)	Reference	
	Dominant model	GG	147 (30.00)	133 (27.20)	0.332	0.962 (0.888, 1.041)
		TT+GT	343 (70.00)	356 (72.80)	Reference	
	Recessive model	TT	108 (22.04)	102 (20.86)	0.652	1.057 (0.831, 1.343)
		GG+GT	382 (77.96)	387 (79.14)	Reference	
rs3829251	Genotypes	AA	48 (9.80)	42 (8.59)	0.363	0.811 (0.516, 1.274)
		GA	215 (4.88)	202 (41.31)	0.302	0.871 (0.669, 1.133)
		GG	227 (46.33)	245 (50.10)	Reference	
	Alleles	A	311 (31.73)	286 (29.24)	0.231	0.965 (0.910, 1.023)
		G	669 (68.27)	692 (70.76)	Reference	
	Dominant model	GG	227 (46.33)	245 (50.10)	0.237	1.076 (0.953, 1.214)
		AA+GA	263 (53.67)	244 (49.90)	Reference	
	Recessive model	AA	48 (9.80)	42 (8.59)	0.513	0.987 (0.948, 1.027)
		GG+GA	442 (90.20)	447 (91.41)	Reference	

*The *P* values are not corrected for multiple testing, Bonferroni corrected *P* = 0.0167 (0.05/3)

As illustrated in Table 1, there were no significant differences in allele, genotype distributions of the *CYP24A1* rs2248359, rs2296241, rs927650, and rs6013897 polymorphisms between the PTB patients and controls (all *P* values >0.05). In addition, we did not detect significant associations between *CYP27A1* rs17470271, rs933994, *CYP27B1* rs4646536, *CYP2R1* rs12794714, rs10741657, rs7935792, rs1562902, and *DHCR7* rs12785878, rs3829251 polymorphisms and the risk of PTB (all *P* values >0.05).

### Association of vitamin D pathway gene SNPs with several clinical features among patients with PTB

We conducted a case-only analysis to explore the potential associations between the genotype, allele frequencies of *CYP24A1*, *CYP27A1*, *CYP27B1*, *CYP2R1*, *GC*, *DHCR7* genes, and several common clinical features among PTB patients (Table S1). *CYP27A1*, *GC*, and *DHCR7* genes polymorphisms were found to be significantly associated with the clinical features. For *CYP27A1* gene, the rs17470271 T allele frequency was significantly lower with leukopenia (*P* = 0.039), and the rs933994 T allele frequency was significantly decreased with drug resistance (*P* = 0.047) (Table 2).

**Table 2:** The positive findings of the associations between vitamin D pathway genes polymorphisms and clinical features of PTB patients

SNP	Allele	Clinical features	Group	Genetypes *n* (%)			*P* value	Alleles *n* (%)		*P* value
	(M/m)			MM	Mm	mm		*M*	*m*	
*CYP27A1*										
rs17470271	A/T	Leukopenia	+	32 (96.97)	1 (3.03)	0	0.106	65 (98.48)	1 (1.52)	**0.039**
			−	374 (82.93)	75 (16.63)	2 (0.44)		823 (91.24)	79 (8.76)	
rs933994	C/T	Drug resistance	+	59 (78.67)	15 (20.00)	1 (1.33)	0.149	133 (88.67)	17 (11.33)	**0.047**
			−	276 (67.81)	116 (28.50)	15 (3.69)		668 (82.03)	146 (17.94)	
*GC*										
rs7041	A/C	Pulmonary infection	+	57 (53.77)	39 (36.79)	10 (9.43)	**0.031**	153 (72.17)	59 (27.83)	**0.050**
			−	229 (60.58)	136 (35.98)	13 (3.44)		594 (78.57)	162 (21.43)	
rs3733359	G/A	Fever	+	34 (39.08)	40 (45.98)	13 (14.94)	0.094	108 (62.07)	66 (37.93)	**0.027**
			−	198 (50.38)	159 (40.46)	36 (9.16)		555 (70.61)	231 (29.39)	
rs16847024	C/T	Fever	+	59 (67.82)	23 (26.44)	5 (5.75)	**0.036**	141 (81.03)	33 (18.97)	**0.009**
			−	310 (78.88)	75 (19.08)	8 (2.04)		695 (88.42)	91 (11.58)	
rs16847024	C/T	DILI	+	48 (68.57)	18 (25.71)	4 (5.71)	0.092	114 (81.42)	26 (18.58)	**0.027**
			−	325 (78.50)	80 (19.32)	9 (2.17)		730 (88.16)	98 (11.84)	
*DHCR7*										
rs12785878	G/T	Pulmonary infection	+	42 (39.62)	45 (42.45)	19 (17.92)	**0.047**	129 (60.85)	83 (39.15)	**0.024**
			−	103 (27.25)	188 (49.74)	87 (23.01)		394 (52.12)	362 (47.88)	

In *GC* gene, the CC genotype and C allele frequencies of rs7041 variant were significantly associated with the increased risk of pulmonary infection (*P* = 0.031, *P* = 0.050, respectively), and the rs3733359 A allele frequency was significantly higher with fever (*P* = 0.027). In addition, the TT genotype and T allele frequencies of rs16847024 were significantly increased with fever (*P* = 0.036, *P* = 0.009, respectively), and the elevated frequency of rs16847024 T allele was associated with DILI (*P* = 0.027). For *DHCR7* gene, rs12785878 TT genotype and T allele frequencies were both significantly decreased with pulmonary infection (*P* = 0.047, *P* = 0.024, respectively). There was no significant relationship between *CYP24A1*, *CYP27B1*, *CYP2R1* genes SNPs and any clinical features.

### Haplotype analysis

We used the SHEsis software to detect the haplotype of *CYP24A1*, *CYP27A1*, *CYP27B1*, *CYP2R1*, *GC*, *DHCR7* gene, and investigated the associations of these haplotypes with PTB susceptibility. Seven main haplotypes (CACT, CGCA, CGCT, CGTT, TACA, TACT, TATT) for *CYP24A1*, three main haplotypes (AC, AT, TT) for *CYP27A1*, five main haplotypes (AGAT, GAAC, GGAT, GGCC, GGCT) for *CYP2R1*, six main haplotypes (AACG, AATG, AGCG, AGCT, CACG, CGCG) for *GC*, and three main haplotypes (GA, GG, TG) for *DHCR7* were detected.

The frequency distributions of these haplotypes among the PTB patients and controls are summarized in Tables 3, 4, 5, 6, and 7. The results demonstrated that the frequencies of *CYP24A1* TACT and *CYP2R1* GGCT haplotypes were significantly higher in the PTB patients than controls (*OR* = 1.25, 95% CI: 1.01-1.56, *P* = 0.039; *OR* = 1.46, 95% CI: 1.05-2.01, *P* = 0.023). Moreover, the frequencies of *CYP2R1* GGAT and *GC* AATG haplotypes were found to be reduced in the PTB patients compared with controls (*OR* = 0.72, 95% CI: 0.54-0.95, *P* = 0.021; *OR* = 0.71, 95% CI:0.53-0.94, *P* = 0.016).

**Table 3:** Haplotype analysis of *CYP24A1* gene in PTB patients and controls

Haplotype	PTB patients [*n* (%)]	Controls [*n* (%)]	*P* value	OR (95% CI)
rs2248359-rs2296241-rs927650-rs6013897				
CACT	27.92 (2.8)	38.05 (3.9)	0.211	0.729 (0.443, 1.199)
CGCA	65.38 (6.7)	60.37 (6.2)	0.623	1.095 (0.762, 1.574)
CGCT	294.59 (30.1)	323.30 (33.1)	0.178	0.875 (0.720, 1.063)
CGTT	170.10 (17.4)	150.40 (15.4)	0.206	1.169 (0.918, 1.489)
TACA	48.89 (5.0)	62.79 (6.4)	1.183	0.770 (0.523, 1.132)
TACT	240.09 (24.5)	203.40 (20.8)	0.039	1.254 (1.012, 1.555)
TATT	49.78 (5.1)	62.97 (6.4)	0.210	0.783 (0.533, 1.149)

Global *χ2* is 10.598, df=6 (frequency<0.03 in both control & case has been dropped)

Fisher’s *p* value is 0.102

**Table 4:** Haplotype analysis of *CYP27A1* gene in PTB patients and controls

Haplotype	PTB patients [*n* (%)]	Controls [*n* (%)]	*P* value	OR (95% CI)
rs17470271-rs933994				
AC	815.00 (83.2)	834.00 (85.3)	0.2	0.853 (0.669, 1.088)
AT	83.00 (8.5)	64.00 (6.5)	0.106	1.321 (0.942, 1.854)
TT	82.00 (8.4)	80.00 (8.2)	0.88	1.025 (0.743, 1.414)

Global *χ2* is 2.697, df=2 (frequency<0.03 in both control and case has been dropped)

Fisher’s *p* value is 0.260

**Table 5:** Haplotype analysis of *CYP2R1* gene in PTB patients and controls

Haplotype	PTB patients [*n* (%)]	Controls [*n* (%)]	*P* value	OR (95% CI)
rs12794714-rs10741657-rs7935792-rs1562902				
AGAT	364.57 (37.2)	347.56 (35.5)	0.381	1.087 (0.902, 1.311)
GAAC	340.32 (34.7)	349.69 (35.8)	0.699	0.964 (0.798, 1.163)
GGAT	92.51 (9.4)	124.85 (12.8)	**0.021**	0.715 (0.538, 0.952)
GGCC	33.40 (3.4)	39.71 (4.1)	0.461	0.838 (0.524, 1.341)
GGCT	95.74 (9.8)	68.28 (7.0)	**0.023**	1.455 (1.051, 2.013)

Global *χ2* is 10.485, df=5 (frequency<0.03 in both control and case has been dropped)

Fisher’s *p* value is 0.033

**Table 6:** Haplotype analysis of *GC* gene in PTB patients and controls

Haplotype	PTB patients [*n* (%)]	Controls [*n* (%)]	*P* value	OR (95% CI)
rs7041-rs3733359-rs16847024-rs4588				
AACG	123.94 (12.6)	122.66 (12.5)	0.916	1.014 (0.776, 1.326)
AATG	93.08 (9.5)	126.88 (13.0)	**0.016**	0.706 (0.531, 0.938)
AGCG	198.01 (20.2)	*165.20* (*16.9*)	0.053	1.255 (0.997, 1.579)
AGCT	317.50 (32.4)	296.95 (30.4)	0.297	1.108 (0.914, 1.343)
CACG	47.17 (4.8)	65.29 (6.7)	0.080	0.709 (0.482, 1.044)
CGCG	160.45 (16.4)	165.10 (16.9)	0.792	0.968 (0.763, 1.230)

Global *χ2* is 11.832, df=5 (frequency<0.03 in both control and case has been dropped)

Fisher’s *p* value is 0.037

**Table 7:** Haplotype analysis of *DHCR7* gene in PTB patients and controls

Haplotype	PTB patients [*n* (%)]	Controls [*n* (%)]	*P* value	OR (95% CI)
rs12785878-rs3829251				
GA	303.25 (30.9)	281.97 (28.8)	0.281	1.112 (0.916, 1.350)
GG	225.75 (23.0)	238.03 (24.3)	0.528	0.935 (0.759, 1.152)
TG	443.25 (45.2)	453.97 (46.4)	0.652	0.960 (0.803, 1.147)

Global *χ2* is 1.225, df=2 (frequency<0.03 in both control and case has been dropped)

Fisher’s *p* value is 0.542

## Discussion

At present, a wide range of research interests have been focused on the non-skeletal metabolism of vitamin D. Vitamin D could stimulate innate immunity during MTB infection, thereby controlling MTB proliferation in macrophages [24, 25]. It has also been reported to be involved in regulating the host cytotoxic T lymphocyte response and the differentiation of natural T cells into regulatory T cells, including the potential role of adaptive immunity during infection [26, 27]. Thus, some researchers speculated that vitamin D deficiency may have a causal role in increasing susceptibility to PTB. In addition, vitamin D deficiency was common in the PTB patients in Chinese population [24]. Previous studies had shown that the genetic variants of vitamin D pathway genes were correlated with the serum 25(OH)D level [28, 29]. The existing studies have concentrated only one or two vitamin D related genes in Chinese population. The association between vitamin D pathway genes polymorphisms and PTB should be further explored; this study is the first study to examine the association between 17 SNPs of vitamin D metabolic pathway genes (*CYP24A1*, *CYP27A1*, *CYP27B1*, *CYP2R1*, *GC*, *DHCR7*) and PTB susceptibility in the Chinese population.

The *CYP24A1* (25-Hydroxyvitamin D-24-hydroxylase), *CYP27A1* (cytochrome P450, family 27, subfamily A, polypeptide 1), *CYP27B1* (25-Hydroxyvitamin D-1a-hydroxylase), *CYP2R1* (vitamin D-25-hydroxylase), *GC* (vitamin D-binding protein, VDBP), and *DHCR7* (7-dehydrocholesterol reductase) are both important genes involved in vitamin D pathway [30, 31]. In the vitamin D pathway, the DHCR7 converts 7-dehydrocholesterol to cholesterol, thus removing the cholesterol pathway from the vitamin D3 synthetic pathway. The previtamin D3 is converted to vitamin D3 in turn with a heat dependent process. As the first step, 25-hydroxylation occurred mainly in the liver, and is regulated primarily by CYP2R1 and CYP27A1. The 25(OH)D is transported to the kidneys by binding to GC, and converted through CYP27B1 to 1,25(OH)2D3 (calcitriol), which is the biologically active form. CYP24A1 could catabolize 25(OH)D, and calcitriol into biologically inactive, water-soluble metabolites for excretion in bile. These genes play important roles in vitamin D metabolism.

Sadykov et al. investigated the genetic variation of vitamin D metabolic pathway gene of the PTB patients in Kazakhstan population, and the results suggested that *CYP24A1* rs6013897 variant was associated with PTB, and the interaction of *CYP24A1* and *VDR* genetic variation might affect PTB susceptibility [22]. However, our study did not support their findings and we found no significant association of rs6013897 variant with PTB susceptibility. Furthermore, the rs2248359, rs2296241, and rs927650 polymorphisms of *CYP24A1* gene were also not contributed to PTB development. In addition to race, the differences in results could be due to the different sample sizes of the two studies, and Sadykov et al. only included an insufficient sample for genetic analysis which might influenced the statistical power of this study. Haplotype analysis demonstrated that *CYP24A1* TACT frequency was related to the increased risk of PTB. This indicated that *CYP24A1* gene polymorphism may be involved in the pathogenesis of PTB, but its precise role remains to be further explored.

Polymorphisms in *CYP2R1* gene could influence vitamin D status in the general population [28], and *CYP27B1* rs4646536 T allele has been shown to be associated with vitamin D deficiency in a family-based study [32]. Moreover, there was an interaction between the *CYP27A1* methylation level and serum 1,25(OH)2D level which was associated with the increased risk of PTB [11]. Thereby, we explored the role of *CYP27A1* rs17470271, rs933994, *CYP27B1* rs4646536, *CYP2R1* rs12794714, rs10741657, rs7935792, and rs1562902 variants in the development of PTB, and no significant result was found. Similarly, a previous study showed that *CYP2R1* gene polymorphism was not associated with susceptibility to TB in Pakistan [9]. Sadykov et al. reported that *CYP2R1*, *CYP27A1*, and *CYP27B1* genetic variation did not affect PTB polymorphism [22]. We also investigated the possible association between these SNPs and clinical features of the PTB patients, and found that *CYP27A1* rs17470271 T allele and rs933994 T allele frequencies were significantly associated with leukopenia, drug resistance in PTB patients, respectively. The emergence of drug resistance was a very noteworthy problem in the treatment of PTB, which would bring adverse effects to the treatment process. This suggests that *CYP27A1* rs933994 might be used as a predictor of drug resistance in patients with PTB, and rs17470271 could also be considered as an indicator of the progression of PTB. Further research is needed to verify these hypotheses. Our results also suggested that *CYP2R1* GGCT haplotype was significantly higher in patients with PTB than in normal controls, while *CYP2R1* GGAT haplotype was significantly reduced. These results are very helpful to further understand the mechanism of *CYP2R1* gene variation in the pathogenesis of PTB.

Vitamin D is circulated in the blood in protein in combination with VDBP, which is encoded by *GC* gene, and albumin. The decreased VDBP mRNA expression level along with significantly lower serum albumin level were found in active PTB patients compared to healthy controls, implying that VDBP, albumin deficiency could play a role in vitamin D deficiency states [33]. Moreover, the genetic variation of *GC* differs in their affinity for vitamin D metabolites in the circulation that could modulate antimycobacterial immunity [34]. Previous studies suggested that the rs7041 and rs4588 polymorphisms were not associated with PTB susceptibility [9, 22]. Our data also confirmed that rs7041 had no effect on PTB susceptibility, while rs4588 mutation was involved in the pathogenesis of PTB. In addition, our results demonstrated that rs3733359 A, rs16847024 T allele frequencies, and AATG haplotype were reduced in PTB patients compared with controls, and these SNPs might contribute to decreased susceptibility to PTB in the Chinese population. Similarly, the variations of *GC* rs3733359 and rs16847024 have also been proved to be the candidate susceptibility markers of gestational diabetes mellitus in Chinese women [35, 36]. In this study, the results showed that the rs7041, rs3733359, and rs16847024 were related to the occurrence of several clinical manifestations, including fever and DILI, pulmonary infection in patients with PTB. This confirmed the important role of *GC* gene variation in the development of PTB. Similar to the results of that study by Sadykov et al. [22], we did not find a significant association between the *DHCR7* variant and the risk of PTB. It is worth noting that the significant association between *DHCR7* rs12785878 and pulmonary infection, possibly suggesting a potential association between *DHCR7* and PTB susceptibility.

Our study has some limitations. Firstly, the study did not exclude the potential influence of some confounding factors, such as treatment regimen, and diet*.* Secondly, the sample size might not be sufficient. Therefore, replication studies with larger sample size in different ethnic groups should be considered to further verify and explore the role of vitamin D metabolic pathways in PTB development.

## Conclusion

Our study provided the evidence that *GC* rs3733359, rs16847024, and rs4588 variant might contribute to PTB susceptibility, while *CYP24A1*, *CYP27A1*, *CYP27B1*, *CYP2R1*, and *DHCR7* genetic variations were not associated with the susceptibility to PTB. Moreover, several SNPs in *CYP27A1*, *GC*, and *DHCR7* genes were related to multiple clinical features, including leukopenia, drug resistance, pulmonary infection, fever, and DILI, in PTB patients.

## Supplementary Information

**Additional file 1: Table S1.** The associations between vitamin D pathway gene polymorphisms and clinical features of PTB patients.

## Data Availability

The data generated and analyzed by this study are available from the corresponding author on reasonable request.
