# Supplementary material for: Association of vitamin D pathway genes polymorphisms with pulmonary tuberculosis susceptibility in a Chinese population
Source: Genes Nutr. 2021 Apr 21;16:6. doi: 10.1186/s12263-021-00687-3 (PMC8061222; doi:10.1186/s12263-021-00687-3)
Supplement: Supplementary file 1 — Additional file 1: Table S1. The associations between vitamin D pathway gene polymorphisms and clinical features of PTB patients. [file 12263_2021_687_MOESM1_ESM.doc]

**Table S1**  The associations between vitamin D pathway gene polymorphisms and

clinical features of PTB patients

| SNP | Allele | Clinical features | Group | Genetypes n (%) | | | P value | Alleles n (%) | | P value |
| --- | --- | --- | --- | --- | --- | --- | --- | --- | --- | --- |
|  | (M/m) |  |  | MM | Mm | mm |  | M | m |  |
| *CYP24A1* |  | | | | | | | | | |
| rs2248359 | C/T | fever | + | 31 | 44 | 12 | 0.893 | 106 | 68 | 0.971 |
|  |  |  | - | 146 | 188 | 59 |  | 480 | 306 |  |
|  |  | drug resistance | + | 23 | 44 | 8 | 0.130 | 90 | 60 | 0.786 |
|  |  |  | - | 155 | 188 | 64 |  | 498 | 316 |  |
|  |  | DILI | + | 22 | 39 | 9 | 0.390 | 83 | 57 | 0.643 |
|  |  |  | - | 157 | 194 | 63 |  | 508 | 320 |  |
|  |  | pulmonary infection | + | 48 | 44 | 14 | 0.134 | 140 | 72 | 0.092 |
|  |  |  | - | 131 | 189 | 58 |  | 451 | 305 |  |
|  |  | leukopenia | + | 12 | 18 | 3 | 0.576 | 42 | 24 | 0.656 |
|  |  |  | - | 167 | 215 | 69 |  | 549 | 353 |  |
|  |  | sputum smear | + | 47 | 63 | 21 | 0.898 | 157 | 105 | 0.667 |
|  |  |  | - | 114 | 147 | 44 |  | 375 | 235 |  |
| rs2296241 | G/A | fever | + | 31 | 44 | 12 | 0.753 | 106 | 68 | 0.521 |
|  |  |  | - | 132 | 194 | 67 |  | 458 | 328 |  |
|  |  | drug resistance | + | 23 | 44 | 8 | 0.164 | 90 | 60 | 0.707 |
|  |  |  | - | 140 | 195 | 72 |  | 475 | 339 |  |
|  |  | DILI | + | 24 | 36 | 10 | 0.857 | 84 | 56 | 0.731 |
|  |  |  | - | 140 | 204 | 70 |  | 484 | 344 |  |
|  |  | pulmonary infection | + | 39 | 53 | 14 | 0.535 | 131 | 81 | 0.297 |
|  |  |  | - | 125 | 187 | 66 |  | 437 | 319 |  |
|  |  | leukopenia | + | 11 | 19 | 3 | 0.439 | 41 | 25 | 0.556 |
|  |  |  | - | 153 | 221 | 77 |  | 527 | 375 |  |
|  |  | sputum smear | + | 39 | 75 | 17 | 0.095 | 153 | 109 | 0.993 |
|  |  |  | - | 108 | 141 | 56 |  | 357 | 253 |  |
| rs927650 | C/T | fever | + | 54 | 26 | 7 | 0.243 | 134 | 40 | 0.238 |
|  |  |  | - | 208 | 155 | 30 |  | 571 | 215 |  |
|  |  | drug resistance | + | 35 | 31 | 9 | 0.176 | 101 | 49 | 0.065 |
|  |  |  | - | 228 | 151 | 28 |  | 607 | 207 |  |
|  |  | DILI | + | 36 | 30 | 4 | 0.578 | 102 | 38 | 0.864 |
|  |  |  | - | 228 | 153 | 33 |  | 609 | 219 |  |
|  |  | pulmonary infection | + | 56 | 44 | 6 | 0.532 | 156 | 56 | 0.060 |
|  |  |  | - | 208 | 139 | 31 |  | 555 | 201 |  |
|  |  | leukopenia | + | 22 | 9 | 2 | 0.348 | 53 | 13 | 0.192 |
|  |  |  | - | 242 | 174 | 35 |  | 658 | 244 |  |
|  |  | sputum smear | + | 70 | 50 | 11 | 0.845 | 190 | 72 | 0.557 |
|  |  |  | - | 171 | 112 | 22 |  | 454 | 156 |  |
| rs6013897 | T/A | fever | + | 65 | 21 | 1 | 0.708 | 151 | 23 | 0.419 |
|  |  |  | - | 277 | 109 | 7 |  | 663 | 123 |  |
|  |  | drug resistance | + | 50 | 23 | 2 | 0.551 | 123 | 27 | 0.308 |
|  |  |  | - | 293 | 108 | 6 |  | 694 | 120 |  |
|  |  | DILI | + | 49 | 20 | 1 | 0.957 | 118 | 22 | 0.192 |
|  |  |  | - | 295 | 112 | 7 |  | 702 | 126 |  |
|  |  | pulmonary infection | + | 74 | 30 | 2 | 0.938 | 178 | 34 | 0.557 |
|  |  |  | - | 270 | 102 | 6 |  | 642 | 114 |  |
|  |  | leukopenia | + | 21 | 11 | 1 | 0.560 | 53 | 13 | 0.303 |
|  |  |  | - | 323 | 121 | 7 |  | 767 | 135 |  |
|  |  | sputum smear | + | 96 | 31 | 4 | 0.091 | 223 | 39 | 0.500 |
|  |  |  | - | 206 | 96 | 3 |  | 508 | 102 |  |
| *CYP27A1* |  | | | | | | | | | |
| rs17470271 | A/T | fever | + | 74 | 12 | 1 | 0.434 | 160 | 14 | 0.880 |
|  |  |  | - | 328 | 64 | 1 |  | 720 | 66 |  |
|  |  | drug resistance | + | 67 | 8 | 0 | 0.340 | 142 | 8 | 0.152 |
|  |  |  | - | 337 | 68 | 2 |  | 742 | 72 |  |
|  |  | DILI | + | 62 | 8 | 0 | 0.471 | 132 | 8 | 0.236 |
|  |  |  | - | 344 | 68 | 2 |  | 756 | 72 |  |
|  |  | pulmonary infection | + | 90 | 16 | 0 | 0.737 | 196 | 16 | 0.668 |
|  |  |  | - | 316 | 60 | 2 |  | 692 | 64 |  |
|  |  | leukopenia | + | 32 | 1 | 0 | 0.106 | 65 | 1 | 0.039 |
|  |  |  | - | 374 | 75 | 2 |  | 823 | 79 |  |
|  |  | sputum smear | + | 111 | 19 | 1 | 0.738 | 241 | 21 | 0.803 |
|  |  |  | - | 254 | 50 | 1 |  | 558 | 52 |  |
| rs933994 | C/T | fever | + | 63 | 23 | 1 | 0.429 | 149 | 25 | 0.311 |
|  |  |  | - | 270 | 108 | 15 |  | 648 | 138 |  |
|  |  | drug resistance | + | 59 | 15 | 1 | 0.149 | 133 | 17 | 0.047 |
|  |  |  | - | 276 | 116 | 15 |  | 668 | 146 |  |
|  |  | DILI | + | 54 | 14 | 2 | 0.329 | 122 | 18 | 0.173 |
|  |  |  | - | 283 | 117 | 14 |  | 683 | 145 |  |
|  |  | pulmonary infection | + | 73 | 32 | 1 | 0.248 | 178 | 34 | 0.724 |
|  |  |  | - | 264 | 99 | 15 |  | 627 | 129 |  |
|  |  | leukopenia | + | 28 | 4 | 1 | 0.128 | 60 | 6 | 0.081 |
|  |  |  | - | 309 | 127 | 15 |  | 745 | 157 |  |
|  |  | sputum smear | + | 94 | 33 | 4 | 0.720 | 221 | 41 | 0.460 |
|  |  |  | - | 207 | 88 | 10 |  | 502 | 108 |  |
| *CYP27B1* |  | | | | | | | | | |
| rs4646536 | G/A | fever | + | 27 | 46 | 14 | 0.149 | 100 | 74 | 0.066 |
|  |  |  | - | 166 | 178 | 49 |  | 510 | 276 |  |
|  |  | drug resistance | + | 35 | 33 | 7 | 0.341 | 103 | 47 | 0.144 |
|  |  |  | - | 158 | 192 | 57 |  | 508 | 306 |  |
|  |  | DILI | + | 29 | 34 | 7 | 0.689 | 92 | 48 | 0.562 |
|  |  |  | - | 166 | 191 | 57 |  | 523 | 305 |  |
|  |  | pulmonary infection | + | 34 | 55 | 17 | 0.140 | 123 | 89 | 0.059 |
|  |  |  | - | 161 | 170 | 47 |  | 492 | 264 |  |
|  |  | leukopenia | + | 11 | 18 | 4 | 0.621 | 40 | 26 | 0.609 |
|  |  |  | - | 184 | 207 | 60 |  | 575 | 327 |  |
|  |  | sputum smear | + | 55 | 61 | 15 | 0.668 | 171 | 91 | 0.379 |
|  |  |  | - | 117 | 145 | 43 |  | 379 | 231 |  |
| *CYP2R1* | | | | | | | | | | |
| rs12794714 | G/A | fever | + | 27 | 47 | 13 | 0.410 | 101 | 73 | 0.264 |
|  |  |  | - | 152 | 188 | 53 |  | 492 | 294 |  |
|  |  | drug resistance | + | 28 | 40 | 7 | 0.424 | 96 | 54 | 0.532 |
|  |  |  | - | 152 | 195 | 60 |  | 499 | 315 |  |
|  |  | DILI | + | 25 | 36 | 9 | 0.872 | 86 | 54 | 0.905 |
|  |  |  | - | 157 | 199 | 58 |  | 513 | 315 |  |
|  |  | pulmonary infection | + | 46 | 43 | 17 | 0.177 | 135 | 77 | 0.542 |
|  |  |  | - | 136 | 192 | 50 |  | 464 | 292 |  |
|  |  | leukopenia | + | 10 | 16 | 7 | 0.388 | 36 | 30 | 0.204 |
|  |  |  | - | 172 | 219 | 60 |  | 563 | 339 |  |
|  |  | sputum smear | + | 46 | 68 | 17 | 0.738 | 160 | 102 | 0.874 |
|  |  |  | - | 115 | 146 | 44 |  | 376 | 234 |  |
| rs10741657 | G/A | fever | + | 40 | 37 | 10 | 0.495 | 117 | 57 | 0.225 |
|  |  |  | - | 158 | 174 | 61 |  | 490 | 296 |  |
|  |  | drug resistance | + | 29 | 36 | 10 | 0.722 | 94 | 56 | 0.843 |
|  |  |  | - | 171 | 175 | 61 |  | 517 | 297 |  |
|  |  | DILI | + | 26 | 35 | 9 | 0.550 | 87 | 53 | 0.753 |
|  |  |  | - | 174 | 178 | 62 |  | 526 | 302 |  |
|  |  | pulmonary infection | + | 47 | 45 | 14 | 0.751 | 139 | 73 | 0.444 |
|  |  |  | - | 153 | 168 | 57 |  | 474 | 282 |  |
|  |  | leukopenia | + | 18 | 9 | 6 | 0.132 | 45 | 21 | 0.396 |
|  |  |  | - | 182 | 204 | 65 |  | 568 | 334 |  |
|  |  | sputum smear | + | 49 | 63 | 19 | 0.591 | 161 | 101 | 0.403 |
|  |  |  | - | 130 | 133 | 42 |  | 393 | 217 |  |
| rs7935792 | A/C | fever | + | 67 | 18 | 2 | 0.898 | 152 | 22 | 0.767 |
|  |  |  | - | 295 | 90 | 8 |  | 680 | 106 |  |
|  |  | drug resistance | + | 52 | 21 | 2 | 0.425 | 125 | 25 | 0.198 |
|  |  |  | - | 311 | 88 | 8 |  | 710 | 104 |  |
|  |  | DILI | + | 52 | 18 | 0 | 0.354 | 122 | 18 | 0.860 |
|  |  |  | - | 313 | 91 | 10 |  | 717 | 111 |  |
|  |  | pulmonary infection | + | 80 | 22 | 4 | 0.349 | 182 | 30 | 0.689 |
|  |  |  | - | 285 | 87 | 6 |  | 657 | 99 |  |
|  |  | leukopenia | + | 21 | 10 | 2 | 0.114 | 52 | 14 | 0.051 |
|  |  |  | - | 344 | 99 | 8 |  | 787 | 115 |  |
|  |  | sputum smear | + | 99 | 30 | 2 | 0.782 | 228 | 34 | 0.706 |
|  |  |  | - | 228 | 69 | 8 |  | 525 | 85 |  |
| rs1562902 | T/C | fever | + | 29 | 47 | 11 | 0.417 | 105 | 69 | 0.473 |
|  |  |  | - | 130 | 191 | 72 |  | 451 | 335 |  |
|  |  | drug resistance | + | 24 | 38 | 13 | 0.959 | 86 | 64 | 0.838 |
|  |  |  | - | 137 | 200 | 70 |  | 474 | 340 |  |
|  |  | DILI | + | 25 | 33 | 12 | 0.884 | 83 | 57 | 0.750 |
|  |  |  | - | 136 | 207 | 71 |  | 479 | 349 |  |
|  |  | pulmonary infection | + | 34 | 54 | 18 | 0.946 | 122 | 90 | 0.865 |
|  |  |  | - | 127 | 186 | 65 |  | 440 | 316 |  |
|  |  | leukopenia | + | 16 | 10 | 7 | 0.065 | 42 | 24 | 0.341 |
|  |  |  | - | 145 | 230 | 76 |  | 520 | 382 |  |
|  |  | sputum smear | + | 37 | 71 | 23 | 0.526 | 145 | 117 | 0.359 |
|  |  |  | - | 103 | 152 | 50 |  | 358 | 252 |  |
| *GC* |  | | | | | | | | | |
| rs7041 | A/C | fever | + | 54 | 30 | 3 | 0.747 | 138 | 36 | 0.482 |
|  |  |  | - | 231 | 142 | 20 |  | 604 | 182 |  |
|  |  | drug resistance | + | 48 | 22 | 5 | 0.341 | 118 | 32 | 0.636 |
|  |  |  | - | 237 | 152 | 18 |  | 626 | 188 |  |
|  |  | DILI | + | 49 | 18 | 3 | 0.125 | 116 | 24 | 0.083 |
|  |  |  | - | 237 | 157 | 20 |  | 631 | 197 |  |
|  |  | pulmonary infection | + | 57 | 39 | 10 | 0.031 | 153 | 59 | 0.050 |
|  |  |  | - | 229 | 136 | 13 |  | 594 | 162 |  |
|  |  | leukopenia | + | 18 | 14 | 1 | 0.694 | 50 | 16 | 0.777 |
|  |  |  | - | 268 | 161 | 22 |  | 697 | 205 |  |
|  |  | sputum smear | + | 79 | 47 | 5 | 0.744 | 205 | 57 | 0.661 |
|  |  |  | - | 181 | 107 | 17 |  | 469 | 141 |  |
| rs3733359 | G/A | fever | + | 34 | 40 | 13 | 0.094 | 108 | 66 | 0.027 |
|  |  |  | - | 198 | 159 | 36 |  | 555 | 231 |  |
|  |  | drug resistance | + | 32 | 34 | 9 | 0.579 | 98 | 52 | 0.307 |
|  |  |  | - | 200 | 166 | 41 |  | 566 | 248 |  |
|  |  | DILI | + | 31 | 27 | 12 | 0.129 | 89 | 51 | 0.140 |
|  |  |  | - | 202 | 174 | 38 |  | 578 | 250 |  |
|  |  | pulmonary infection | + | 54 | 42 | 10 | 0.800 | 150 | 62 | 0.510 |
|  |  |  | - | 179 | 159 | 40 |  | 517 | 239 |  |
|  |  | leukopenia | + | 20 | 11 | 2 | 0.310 | 51 | 15 | 0.128 |
|  |  |  | - | 213 | 190 | 48 |  | 616 | 286 |  |
|  |  | sputum smear | + | 63 | 52 | 16 | 0.443 | 178 | 84 | 0.611 |
|  |  |  | - | 146 | 133 | 26 |  | 425 | 185 |  |
| rs16847024 | C/T | fever | + | 59 | 23 | 5 | 0.036 | 141 | 33 | 0.009 |
|  |  |  | - | 310 | 75 | 8 |  | 695 | 91 |  |
|  |  | drug resistance | + | 59 | 15 | 1 | 0.722 | 133 | 17 | 0.543 |
|  |  |  | - | 312 | 83 | 12 |  | 707 | 107 |  |
|  |  | DILI | + | 48 | 18 | 4 | 0.092 | 114 | 26 | 0.027 |
|  |  |  | - | 325 | 80 | 9 |  | 730 | 98 |  |
|  |  | pulmonary infection | + | 79 | 23 | 4 | 0.653 | 181 | 31 | 0.372 |
|  |  |  | - | 294 | 75 | 9 |  | 663 | 93 |  |
|  |  | leukopenia | + | 28 | 5 | 0 | 0.431 | 61 | 5 | 0.187 |
|  |  |  | - | 345 | 93 | 13 |  | 783 | 119 |  |
|  |  | sputum smear | + | 99 | 28 | 4 | 0.814 | 226 | 36 | 0.512 |
|  |  |  | - | 238 | 60 | 7 |  | 536 | 74 |  |
| rs4588 | G/T | fever | + | 37 | 45 | 5 | 0.455 | 119 | 55 | 0.300 |
|  |  |  | - | 150 | 205 | 38 |  | 505 | 281 |  |
|  |  | drug resistance | + | 35 | 35 | 5 | 0.328 | 105 | 45 | 0.174 |
|  |  |  | - | 154 | 215 | 38 |  | 523 | 291 |  |
|  |  | DILI | + | 24 | 39 | 7 | 0.649 | 87 | 53 | 0.414 |
|  |  |  | - | 166 | 212 | 36 |  | 544 | 284 |  |
|  |  | pulmonary infection | + | 50 | 48 | 8 | 0.168 | 148 | 64 | 0.110 |
|  |  |  | - | 140 | 203 | 35 |  | 483 | 273 |  |
|  |  | leukopenia | + | 13 | 18 | 2 | 0.832 | 44 | 22 | 0.794 |
|  |  |  | - | 177 | 233 | 41 |  | 587 | 315 |  |
|  |  | sputum smear | + | 51 | 72 | 8 | 0.336 | 174 | 88 | 0.300 |
|  |  |  | - | 177 | 156 | 32 |  | 510 | 220 |  |
| *DHCR7* |  | | | | | | | | | |
| rs12785878 | G/T | fever | + | 31 | 37 | 19 | 0.422 | 99 | 75 | 0.442 |
|  |  |  | - | 114 | 194 | 85 |  | 422 | 364 |  |
|  |  | drug resistance | + | 23 | 40 | 12 | 0.392 | 86 | 64 | 0.394 |
|  |  |  | - | 122 | 192 | 93 |  | 436 | 378 |  |
|  |  | DILI | + | 23 | 37 | 10 | 0.250 | 83 | 57 | 0.177 |
|  |  |  | - | 122 | 196 | 96 |  | 440 | 388 |  |
|  |  | pulmonary infection | + | 42 | 45 | 19 | 0.047 | 129 | 83 | 0.024 |
|  |  |  | - | 103 | 188 | 87 |  | 394 | 362 |  |
|  |  | leukopenia | + | 10 | 16 | 7 | 0.995 | 36 | 30 | 0.930 |
|  |  |  | - | 135 | 217 | 99 |  | 487 | 415 |  |
|  |  | sputum smear | + | 38 | 62 | 31 | 0.832 | 138 | 124 | 0.542 |
|  |  |  | - | 95 | 145 | 65 |  | 335 | 275 |  |
| rs3829251 | G/A | fever | + | 37 | 41 | 9 | 0.718 | 115 | 59 | 0.482 |
|  |  |  | - | 186 | 169 | 38 |  | 541 | 245 |  |
|  |  | drug resistance | + | 33 | 33 | 9 | 0.749 | 99 | 51 | 0.499 |
|  |  |  | - | 191 | 178 | 38 |  | 560 | 254 |  |
|  |  | DILI | + | 28 | 34 | 8 | 0.493 | 90 | 50 | 0.259 |
|  |  |  | - | 197 | 178 | 39 |  | 572 | 256 |  |
|  |  | pulmonary infection | + | 47 | 44 | 15 | 0.217 | 138 | 74 | 0.243 |
|  |  |  | - | 178 | 168 | 32 |  | 524 | 232 |  |
|  |  | leukopenia | + | 12 | 15 | 6 | 0.181 | 39 | 27 | 0.092 |
|  |  |  | - | 213 | 197 | 41 |  | 623 | 279 |  |
|  |  | sputum smear | + | 64 | 57 | 10 | 0.484 | 185 | 77 | 0.240 |
|  |  |  | - | 133 | 140 | 32 |  | 406 | 204 |  |
